# A Novel QKD Approach to Enhance IIOT Privacy and Computational Knacks

**DOI:** 10.3390/s22186741

**Published:** 2022-09-06

**Authors:** Kranthi Kumar Singamaneni, Gaurav Dhiman, Sapna Juneja, Ghulam Muhammad, Salman A. AlQahtani, John Zaki

**Affiliations:** 1 Department of Computer Science and Engineering, School of Technology, GITAM Deemed to be University, Visakhapatnam 530045, Andhra Pradesh, India; 2 Department of Electrical and Computer Engineering, Lebanese American University, Beirut 1102 2801, Lebanon; 3 University Centre for Research and Development, Department of Computer Science and Engineering, Chandigarh University, Gharuan, Mohali 140413, Punjab, India; 4 Department of Computer Science and Engineering, Graphic Era Deemed to be University, Dehradun 248002, Uttarakhand, India; 5 KIET Group of Institutions, Delhi NCR, Ghaziabad 201206, Uttar Pradesh, India; 6 Research Chair of New Emerging Technologies and 5G Networks and Beyond, Computer Engineering Department, College of Computer and Information Sciences, King Saud University, Riyadh 11543, Saudi Arabia; 7 Computer Engineering Department, College of Computer and Information Sciences, King Saud University, Riyadh 11543, Saudi Arabia; 8 Department of Computer and Systems, Faculty of Engineering, Mansoura University, Mansoura 35516, Egypt

**Keywords:** chaotic key generator, cybersecurity, IIoT, novel dynamic quantum key distribution algorithm, 5G

## Abstract

The industry-based internet of things (IIoT) describes how IIoT devices enhance and extend their capabilities for production amenities, security, and efficacy. IIoT establishes an enterprise-to-enterprise setup that means industries have several factories and manufacturing units that are dependent on other sectors for their services and products. In this context, individual industries need to share their information with other external sectors in a shared environment which may not be secure. The capability to examine and inspect such large-scale information and perform analytical protection over the large volumes of personal and organizational information demands authentication and confidentiality so that the total data are not endangered after illegal access by hackers and other unauthorized persons. In parallel, these large volumes of confidential industrial data need to be processed within reasonable time for effective deliverables. Currently, there are many mathematical-based symmetric and asymmetric key cryptographic approaches and identity- and attribute-based public key cryptographic approaches that exist to address the abovementioned concerns and limitations such as computational overheads and taking more time for crucial generation as part of the encipherment and decipherment process for large-scale data privacy and security. In addition, the required key for the encipherment and decipherment process may be generated by a third party which may be compromised and lead to man-in-the-middle attacks, brute force attacks, etc. In parallel, there are some other quantum key distribution approaches available to produce keys for the encipherment and decipherment process without the need for a third party. However, there are still some attacks such as photon number splitting attacks and faked state attacks that may be possible with these existing QKD approaches. The primary motivation of our work is to address and avoid such abovementioned existing problems with better and optimal computational overhead for key generation, encipherment, and the decipherment process compared to the existing conventional models. To overcome the existing problems, we proposed a novel dynamic quantum key distribution (QKD) algorithm for critical public infrastructure, which will secure all cyber–physical systems as part of IIoT. In this paper, we used novel multi-state qubit representation to support enhanced dynamic, chaotic quantum key generation with high efficiency and low computational overhead. Our proposed QKD algorithm can create a chaotic set of qubits that act as a part of session-wise dynamic keys used to encipher the IIoT-based large scales of information for secure communication and distribution of sensitive information.

## 1. Introduction

The industrial internet of things (IIoT) has quickly turned out to be an internal module of modern industry-related business, specifically in computerization and data analysis for effective decision-making. The IIoT guarantees advanced enterprise prototypes and standards for numerous business territories with global interconnectivity facilities, economic and efficacy cyber analytical software, and superior decision-making structures designed for the keenness of business. IIoT organizations are at risk because of various cyberattacks on different stages of the interconnectivity and transmission structure. With the intricate characteristics of the IIoT, it is hard to promise authentication, integrity, confidentiality, and availability, which causes cyber data leakage over internetwork communications, apprehensions of perilous multi-organizational structure loss, and conceded cybersecurity, the privacy of netizens and the private information of employees [1]. In this paper, we can provide cybersecurity necessities for a confident and secure IIoT ecology that reaches the standards of current industries such as AWS, Azure IIOT, Oracle IoT Cloud, etc., by incorporating our proposed algorithm, which is a novel chaotic dynamic QKD. We examine potential forthcoming research objectives to increase the cybersecurity, confidentiality, and welfare of IIoT-related industries. Securing confidential data over the internet should incorporate efficient and stringent encipherment practices. In the past, many existing proposed methods had limitations along with their features [2]. Specifically, IIoT appliances had many constraints such as computational speed, limited storage, and network overhead [3]. Currently, several algorithms are available to secure sensitive data over the IIoT infrastructure. One is quantum ciphertext policy attribute-based encryption (QCP-ABE) [4]. QCP-ABE is one of the fastest public-key cryptographic approaches, which requires low computational time to generate a random key based on QKD, encipherment, and decipherment using CP-ABE when compared to numerous existing cryptographical approaches [5,6,7]. One of the most significant advantages of the QCP-ABE system is its chaotic randomized brisk key length which will resolve man-in-the-middle attacks over cyberspace and the rate of encipherment and decipherment compared to KP-ABE, previous versions of CP-ABE, and other mathematical-based public key cryptographic approaches [8,9,10]. The implementation of QCP-ABE depends on fundamental substitutional and transpositional cryptographic techniques along with chaotic functions whose average time complexity is o (*n* log *n*), where *n* is the randomized key length which dynamically varies for every session [11,12,13]. Still, in the QCP-ABE approach, they consider only four states of qubits [14,15,16,17] with reference to four directions of polarizers, i.e., horizontal, vertical, left orthogonal, and right orthogonal states of polarization (SOPs) which produce less key size and may create chances of key prediction using brute force analytical approach on quantum-based supercomputers in the future. IIoT-related internetworks and essentials gradually developed for telecommunications and service providers. In addition, the progress in the usage and deployment of smartphone platforms and mobile applications has been seen as prodigious. Most of the existing IIoT key distribution protocols are not computationally and communicationally lightweight; fail to efficiently knob the addition/revocation of nodes; are not good at fast re-keying; are vulnerable to node capture attacks and server caricature attacks and fail to provide overall confidentiality. In addition, intended users must depend upon a third-party user to produce the key, and the distribution of this key may be compromised and cause man-in-the-middle attacks, brute force attacks, etc. Consequently, this article deliberates the present state of the art of IIoT-related technologies along with the unrestricted safety and privacy necessities achieved with help of the proposed quantum key distribution approach [18,19]. There are several strategies and approaches of QKD that are demonstrated along with the standards and protocols of their usage and previous implementations over IIoT-real networks. Additionally, this article identifies gaps including serious facets of how QKD should be accomplished and efficiently applied to IIoT-real networks through effective and advanced versions of QKD and sensitive information distribution to users in IIoT-real networks [20]. In our proposed novel and advanced QKD approach, we take four more states of qubits along with polarizers which leads to generating a reasonable randomized key size that can altogether avoid most of the cyberattacks on organizational and personal data which may be communicated via IIoT infrastructures, with the same or less computational overhead for key generation, encipherment, and decipherment.

## 2. Related Work

Most IIoT-based cryptosystems should consider many aspects and metrics such as the vital generation time for encipherment and decipherment, the time required for encipherment and decipherment, the space needed for the data storage of users, and the computational time for the data processing of users. Consequently, several scientists involved in designing optimal cryptosystems consider the abovementioned metrics. An ECC-based, lightweight, no pairing, attribute-based encipherment system is anticipated for IIoT grounded apps concerning the reserve constriction component to discourse-protected communication and ciphered text admittance policies upon the cluster-based load-balancing techniques [21]. A mixed encipherment system associated with the advanced encryption standard (AES) and elliptical curve cryptographic approach (ECC) for IIoT-based cybersecurity [22]. A cross-based ciphered process in the public key cryptographic method and single key cryptographic approach setup in one model provides enhanced safety and high speediness, less space for information storage, and the most appropriate IIoT devices with a base-level investigation stage only [23]. A study related numerous practices associated with the IIoT environment based on LB-PKC and its encounters in the IIoT environment since it is not safe from attacks related to quantum. So, the following reports a few of the conventional cryptographical practice insufficiencies. LB-PKC was anticipated for security over attacks based on quantum. Bi-linear polynomial-based (QCP-ABE) cryptography is a novel approach to post-quantum cryptanalytics that is well thought out and appropriate to incorporate beside IIoT devices [24]. To address the secure and scalable problems of the traditional IIoT framework based on blockchain, a deep learning-based algorithm was introduced [25,26]. Tang, Yongli, et al. proposed a cloud-based IIoT PEKS system capable of continuous searching based on multiple users [15]. Moreover, the space required is pointedly condensed related to existing models [27]. Adnan et al. reviewed and discussed the prevailing quantum cryptographical protocols which can be implemented in fifth-generation networks along with their comparative analysis, performance, and efficiency with limitations [28]. De, Rohit, et al. expressed their perceptions of how the DJ (Deutsch–Jozsa) protocol works through this and how the entropy may be enhanced in DJ packets. Additionally, the authors studied various quantum processing and replication with the Google Cirq Python library [29]. Aji et al. investigated and evaluated different latest QKD simulators concerning numerous attributes to enhance the reliability of the overall system to obtain an accurate measurement of qubits [30]. Scarani et al. provided a brief periodical analysis and survey of the practicality of QKD and related speculative simulators, which can measure the chaotic nature of qubits [31]. Renne et al. proposed a methodology that studies the characteristics of typical physical systems for different independence constraints which might not be held. They introduced novel uncertainty metrics known as smooth min and max entropy and considered von Neumann entropy simplifications. Additionally, they developed another kind of quantum-based de Finetti’s interpretation theorem [32]. Shor et al. proved that the BB84 QKD is securely based on entanglement purification, which could be verified for privacy and safety based on the proof of security of Lo and Chau for related protocol approaches [33]. Serna et al. [34] discussed the S13 protocol which is similar to the BB84 standard for key distribution using quantum properties by adding private appeasement after an arbitrary seed and a public cryptographical approach that can generate a larger set of secure key pairs. Liu, Li, et al. [35] applied the reflexive distract state approach to the round-robin differential-phase-shift quantum key distribution standard (RRDPS QKD) by obtaining a novel key production ratio formulation to enhance the key production rate. Pei, Jiaming, et al. [36] used an advanced homomorphic encipherment approach to address the skirmishes among sensitive info distribution and confidentiality preservation for various IIoT environments based on cloud platforms. Here we represented a comparative analysis of existing QKD approaches with our proposed model in Table 1.

## 3. Quantum Cryptographic Models and Issues

Applied cryptographic domains such as the quantum cryptographic field based on quantum physics and mechanics policies for cryptosystem development provide much security compared to traditional cryptographic models [13]. QC is founded on photons, and their underlying essential QC characteristics such as quantum entanglement and quantum key distribution (QKD) are used to build an unbreakable cryptographical system since the way the key is generated and data are transmitted, it is almost impossible to predict the photon state of any system while transmitting data without noticing the intended and authorized person who is part of the system [27,30]. At any time, confidential user data are transmitted via a network from the source to destination and vice versa, some malevolent person may attempt to capture the transmission, and the state of change of one qubit is associated with its replica as per its quantum entanglement property, and the source and destination users are instantly notified by the change in the qubit of the intruder [33,37].

Whatever the user data transmitted using quantum cryptographical- (QC) based systems should not be penetrated by third-party unauthorized persons or intruders [39]. At present, cryptographical systems are applying mathematical principles to build effective cryptographical systems that can break these models, which can be a critical and complex mission concerning classical computers. Still, there may be an evolvement of quantum systems that can easily break the classical mathematical cryptographical approaches within a reasonable time. As a result, researchers and developers are currently starting work on QC approaches rather than traditional mathematical-based cryptosystems specifically on COVID and diabetes-related sensitive bio-medical records in recent days [40,41,42,43,44,45]. We can receive and send confidential user information securely and effectively using QC approaches without cyberattacks such as man-in-the-middle attacks. The origin of QC systems lies in the fact that QC employs the tiniest specific elements present in nature, i.e., qubits, also called photons. Qubits have a peculiar characteristic in that they are not in a single state in every instance. It is impossible to measure their state while in transmission by any existing techniques.

### 3.1. Traditional Quantum Key Distribution (QKD) Methods

A QKD approach is a primary step that we use to implement quantum cryptographical algorithms. We are already familiar with how a group of qubits is used to transmit the confidential information of users by using approaches such as quantum entanglement and state of polarization (SOP) concerning various angles and directions of qubits during transmission. Usually, a qubit has a characteristic known as a state/spin. Each qubit has four states of spins. In general, they are: left orthogonal, right orthogonal, vertical, and horizontal. The left and right orthogonal streams are part of the diagonal SOP, and the vertical and horizontal streams are part of the rectilinear SOP. The SOPs of QKD-related qubits and associated properties are listed in Table 2.

#### 3.1.1. BB84

BB84 was the first quantum key distribution method developed by Brassard and Bennett in 1984 [33,37]. Based on the Heisenberg uncertainty principle, they created the BB84 protocol for securely exchanging sensitive information among source and destination devices. It is the first quantum cryptographical protocol that is demonstrably secure based on the properties given in Table 2. The actual inner position of a qubit is exemplified by ket|ψ⟩, which is a complex numbers-based vector depiction in quantum physics. For the qubit state measurement and results, they used ket|ψ⟩ vector coefficients. By transmitting the qubit via a polarizer, they manipulated the|ψ⟩ coefficients, which led to setting the state of the qubit at some particular angle [18]. They used dual polarizers to filter qubits in the BB84: a diagonal-directed polarizer and a rectilinear-based polarizer. Each polarizer filter|ψ⟩ vector contents were in a randomized set of states: 0° and Π/2° for the rectilinear-based polarizer, and Π/4° and 5Π/4° for the diagonal-directed polarizer. The working principle of these two polarizers is that the state of the qubit can be measured with the matched state of the polarizer while receiving the qubit so that they can generate a random set of matched states which will act as a key for sensitive data encipherment and decipherment.

The working principle of the BB84 is that, for instance, both a conventional and a quantum network link are necessary for the communicators, Singh and King, to share sensitive information. A basic coaxial cable may be sufficient for the standard traditional link, and an optical fiber cable is needed to establish a confidential quantum network link among communicators. Now, both Singh and King use the rectilinear-based polarizers and the diagonal-directed polarizers for the transmission of qubits and to measure the individual instance state of qubits traveled via polarizers through the confidential quantum network link. Communicators use the state of polarization to translate and signify whether qubit values are one or zero at that instance. The BB84 qubit SOP-based encrypting design is presented in Table 3. Afterward, based on the qubit properties, they create a secret key that will help the actual information transmit between the communicators, Singh and King, over the traditional standard channel. Based on Singh and King communicators, the line-by-line BB84 QKD procedure is presented in Table 4.

#### 3.1.2. BB92

Bennett developed a simplified BB84 protocol version in 1992 [33,37]. The primary variation between BB84 and BB92 was that BB84 used four non-orthogonal SOPs for bit-level encoding, whereas BB92 used only two. The two non-orthogonal SOPs of BB92 for bit-level encoding are represented in Table 5. Each SOP base does not have a 0 and 1 binary encoding since in its place, he uses the base as the single binary value. In BB92, the SOP of the qubit was not calculated for the unmatched SOPs, and it only considered matched patterns. Consequently, King can exactly predict the correctly matched measurement of the qubits of Singh. Concerning key size, the BB92 key length is smaller than the BB84 key length since BB92 uses only two non-orthogonal SOPs [38,46].

## 4. Security Issues in IIoT Systems

IIoT gadgets and machinery face many issues and rising constraints concerning the security and privacy of user-sensitive information and the interconnected cyber networks day by day. Traditional IoT and IIoT designs failed to predict unauthorized users or eavesdroppers while communicating [47]. In some scenarios, a single node of the entire IIoT internetwork might be compromised, and nodes or devices of the rest of the whole network might be sending and receiving confidential information through that compromised device up to the time of the compromised device being discovered. A few types of bugs or viruses may affect the devices of the whole IIoT network, and those bugs or viruses can only be eliminated or discarded by restarting all the compromised devices. Still, in general, it is tough to restart or reboot commercial and large-scale machinery for many months or years because of their 24 × 7 working nature in production. Therefore, there is a need for a robust security framework to address all the existing attacks such as man-in-the-middle attacks, Trojan horse attacks, brute force attacks, etc., which IIoT systems are majorly susceptible to. There are few existing solutions to address IIoT security issues via both traditional cryptographic techniques and applied quantum cryptographic techniques which are still vulnerable due to these current methods [48]. Classical computers which are part of IIoT may not crack traditional mathematical-based cryptographic approaches. Still, it is very easy for quantum devices, which are the future of IIoT internetworking, because of their excellent computational capabilities and efficiency compared to classical computers. Of course, the quantum cryptographical approaches are much better than classical cryptographical approaches, but still, the existing quantum techniques such as BB84 and BB92 are not up to the mark to address some of the attacks such as photon numeric split attacks and Trojan horse attacks due to the basis of their limited SOP (max four in BB84 and min two in BB92) and shorter length in key size [33,37]. The majority of conventional IIoT-based key distribution standards failed to dynamically manage the addition and revocation of nodes with the quick, reliable issuing of the keys. In addition, for the distribution of keys, the intended users were dependent upon third-party users which causes man-in-the-middle attacks. Among all the conventional key distribution models, some are not computationally or communicationally lightweight, some are vulnerable to node capture attacks, and some are vulnerable to server caricature attacks. Due to the abovementioned challenges and limitations, the conventional IIoT–key distributed standards fail to provide overall confidentiality [49].

## 5. Proposed Multi-Qubit Quantum Cryptographic Model

We proposed a multi-state qubit QKD model to reduce the computational overhead of dynamic key generation along with QKD security enhancement which will help to discard some of the attacks such as MIMD, photon number splitting (PNS) attacks, and faked state attacks compared to traditional models and provide more security for IIoT applications that desire it. In this model, we particularly consider the octal SOPs of qubit transmission, which can produce a highly chaotic session-wise dynamic list of secret keys for the encipherment and decipherment of the data of users. To implement the proposed standard, we used a set of Java snippets and customs as a graphical representation for the inputs of end-users and to demonstrate results. The persistence of this approach is to virtualize the generation and transmission of qubits using the utmost accurate technique.

In the proposed scheme, King plans to transmit a stream of bits to Singh from multiple sources, for instance, three sources *a*, *b*, and *c* each with a bitstream length of n. King then encodes these three bitstreams as a tensor product of n qubits.

$$\left|\psi \rangle \right.={⊗}^{n}i=1\;|\psi \;a_{i}b_{i}c_{i}\;\rangle$$

where *a_i_*, *b_i_*, and *c_i_* are the *i*th bits of *a*, *b*, and *c*, correspondingly. Collected *a_i_b_i_c_i_* provides a representation of the following eight qubit states:

$$\left|\psi 000\rangle =\right|0\rangle$$

$$\left|\psi 001\rangle =\right|1\rangle$$

$$\left|\psi 010\rangle =\left|+\rangle =1/\surd 2\right|0\rangle +1/\surd 2\right|1\rangle$$

$$\left|\psi 011\rangle =\left|-\rangle =1/\surd 2\;\right|0\rangle \;―1/\surd 2\;\right|1\rangle$$

$$\left|\psi 100\rangle =\left|Ꭓ\rangle =1/2\;\right|0\rangle +1/2\;\right|1\rangle$$

$$\left|\psi 101\rangle =\left|Ӿ\rangle =1/2\;\right|0\rangle ―1/2\;\right|1\rangle$$

$$\left|\psi 110\rangle =\left|Ꭓ\rangle =\surd 3/2\;\right|0\rangle +\surd 3/2\;\right|1\rangle$$

$$\left|\psi 111\rangle =\left|Ӿ\rangle =\surd 3/2\;\right|0\rangle ―\surd 3/2\;\right|1\rangle$$

### 5.1. Multi-Qubit QKD Pseudocode and Simulation

The pseudocode of the proposed model is given below with a sequence of steps such as transmission over the quantum link, transmission over the classical link, unprocessed key mining from various bases, fault tolerant valuation, and the final key production.

Step1: Communication over the quantum channelKing: Generate multi-stream of bits a, b, and c, and each bitstream length of *n*FOR every bit from a multi-bit source S  select randomly from (a,b,c) resulting base S {a[i], b[i], c[i]}END FORFOR every bit from a multi-bit source SProduce a photonIF S[a_i_b_i_c_i_] = 000 and base[i] = RL1 polarize the photon state is either 0° or 180°IF S[a_i_b_i_c_i_] = 001 and base[i] = RL2 polarize the photon state is either 30° or 210°IF S[a_i_b_i_c_i_] = 010 and base[i] = SRL1 polarize the photon state is either 45° or 225°IF S[a_i_b_i_c_i_] = 011 and base[i] = SRL2 polarize the photon state is either 60° or 240°IF S[a_i_b_i_c_i_] = 100 and base[i] = LOG1 polarize the photon state is either 90° or 270°IF S[a_i_b_i_c_i_] = 101 and base[i] = SLOG1 polarize the photon state is either 120° or 300°IF S[a_i_b_i_c_i_] = 110 and base[i] = ROG2 polarize the photon state is either 135° or 315°IF S[a_i_b_i_c_i_] = 111 and base[i] = SROG2 polarize the photon state is either 150° or 330°transmit raw bit stream raw[a_i_b_i_c_i_] to SingEND FORSing:FOR every raw bit stream raw[a_i_b_i_c_i_] obtainedgenerate RANDOM (raw[a_i_b_i_c_i_]) results base^1^[i]measure raw bit stream raw^1^[a_i_b_i_c_i_] with corresponding base^1^[i]finally obtained bit stream is result ^1^[i]END FORStep 2: Communication over the classical channel    2.1: Transmission      FOR every bit stream of result ^1^[i]         transmit base ^1^[i] to King      END FOR    2.2: Key generation from raw bit stream      King:      FOR each bit result ^1^[i]         IF base[i] != base^1^[i]           discard bit result[i] from string S         END IF      END FOR      Sing:      FOR each bit result ^1^[i]         IF base ^1^[i] != base[i]           discard bit result ^1^[i] from string S         END IF      END FORStep 3: Fault-tolerant valuation to predict the level of security    King and Sing:      FOR a group of bits selected randomly from the bit stream S         IF result ^1^[i] = result[i] and base[i] = base ^1^[i]              No or less error rate and snooping                discard result^1^[i] and result[i]         END IF         ELSE              Major error rate and snooping              discard the entire bit stream and retransmit         END ELSE      END FORStep 4: Final key production  Finally filtered bits from the bit streams result ^1^[i] and result[i] after successful execution of IF block of Fault-tolerant valuation is our resultant key.

Experimental setup:

It is easy to generate a qubit object in Java; however, to preserve the confidentiality and integrity of the implementation, some instructions need to be followed. The basic qubit behavior and characteristic is that Singh directs to King and controls King after predicting its SOP. Thus, individual SOPs should be confidential; to achieve this, we use a private access specifier in Java. Using the qubit class constructor, we produce a new qubit object with a standard SOP that acts exactly like a real qubit. For instance,

**private** String sop, base;**public** Qubit(String sop, String base){    **this**.sop = sop; **this**.base = base;}

**Figure 1:**
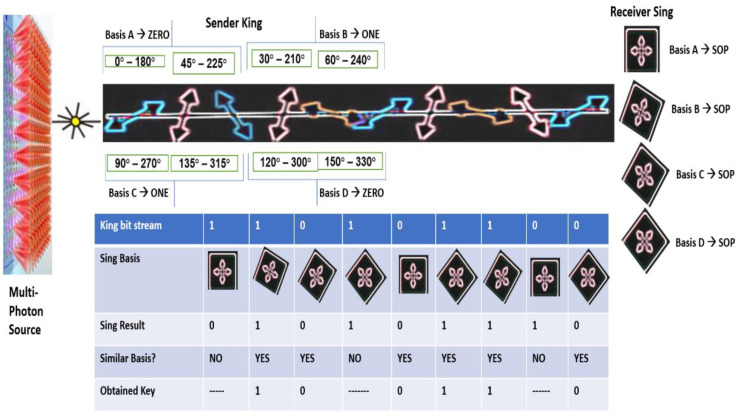
Design and construction of the proposed multi-bit QKD. King uses four conjugated basis to encode an arbitrary stream of bits. Individual bits where King and Singh cast off the identical basis is considered as the obtained final key.

Here, we intentionally put SOP and the base variables as private because the SOP of the qubit of Singh should not be visible to the outside (for instance, in the view of King), and the class qubit can directly access them. We applied the class “Random” in Java for the chaotic production of qubit variant states. We used octa-qubit bases to capture the matched patterns at the side of the receiver. The octal states are represented as {(0°, 180°), (90°, 270°), (45°, 225°), (135°, 315°), (60°, 240°), (120°, 300°), (30°, 210°), (150°, 330°)} to signify the states of the qubits over the randomly directed transmission qubits as shown in Figure 1. The corresponding base representations for the octal states of qubits are given as ‘┼’, ’ X’, ’±’, and ’Ӿ’.

**Figure 2:**
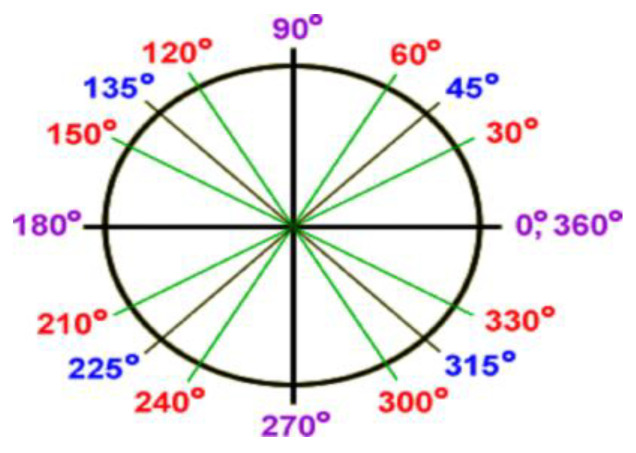
The multi-qubit octal states depiction of the proposed model.

The originator produces the range of values from 0 to 1, which is mapped with the full direction of the qubit; at the receiver side, we need to select the matched base according to our octal state representation. The considerable equivalent random numbers generated as per the movement of the qubit are {0, 0.125, 0.25, 0.375, 0.5, 0.625, 0.75, 0.875, 1} as represented in Figure 2. However, the chaotic producer creates multiple randomized values from 0 to 1, and we set up the nearest values which are matched with the above represented predefined set from 0 to 1, which is private to Singh only as shown in the below snippet:

**public** String createRandomBase(){    **double** base_num = qubit.randomGenerator();    **if** (base_num <0 && base_num <0.25)    base = “±”;    **else if** (base_num >0.25 && base_num<0.5))     base = “┼”;    **else if** (base_num >0.5 && base_num<0.75)     base = “Ӿ”;    **else if** (base_num >0.75 && base_num<1))    base = “X”;    **return** base;}

According to the selected base, the SOP sequence is chaotic. The base values and the SOPs of the above snippet are represented as the polarizer angle base “┼” means to capture only the precise vertical and the horizontally directed matched qubits {(0°, 180°), (90°, 270°)}. The polarizer angle base “X” means to capture only the precise left and right orthogonal-directed matched qubits {(45°, 225°), (135°, 315°)}. Here, we added two more variants to also capture other directions of the qubit. The polarizer angle base “±” means to capture different directions of the qubit in between the straight rectilinear and straight orthogonal positions that are to capture the directed matched qubits {(60°, 240°) (120°, 300°)} of the top half circle. The polarizer angle base “Ӿ” means to capture other directions of the qubit in between the straight rectilinear and straight orthogonal positions that are to capture the directed matched qubits {(30°, 210°) (150°, 330°)} of the bottom half-circle. The entire process is given as:

**public** String CreateSop(String base) {

RL1 = “┼”; SRL1 = “±”;RL2 = “┼”; SRL2 = “±”;LOG1 = “X”; SLOG1 = “Ӿ”;ROG2 = “X”; SROG2 = “Ӿ”;

  **if** (base.equals(*RL1*)|| base.equals(*RL2*))  {     **double** base_num = qubit.randomGenerator();     **if** (base_num >0.25 && base_num <0.5)     sop = “|”;     **else**     sop = “—”;  }  **else if** (base.equals(*LOG1*)|| base.equals(*ROG2*))  {     **double** base_num = qubit.randomGenerator();     **if** (base_num > 0.75 && base_num <1)     sop = “/”;     **else**     sop = “\\”;  }  **else if** (base.equals(S*RL1*)|| base.equals(S*RL2*))  {     **double** base_num = qubit.randomGenerator();     **if** (base_num >0 && base_num < 0.25)     sop = “**±_**|”;     **else**     sop = “**±_**—”;  }  **else if** (base.equals(S*LOG1*)|| base.equals(S*ROG2*))  {     **double** base_num = qubit.randomGenerator();     **if** (base_num > 0.5 && base_num < 0.75)     sop = “ Ӿ_/”;     **else**     sop = “ Ӿ_\\”;  }  **return** sop; }

### 5.2. Multi-Qubit QKD Measurement

The central portion of secure end-to-end transmission by using the proposed QKD technique is the measurement of the multi-qubits of Singh by King. This means, we need to address how King can produce his set of bits; these bits may be a part of the private key. The calculation of the qubits of Singh correspondingly abolishes the generated bits, letting Singh transmit his base set to King only, and access or capture by third-party users or intruders is impossible. The states of the multi-qubits calculation process function through predicting the base of Singh first alongside the base of King. If the chaotic grounds of Singh and King are accorded, the matched pattern bit sets will be used as part of the secret key for the encipherment and decipherment process. In a counter case, the chaotic bases of Singh and King are not accorded. Afterward, the restrained bit sets are discarded. In both cases, the multi-qubit set should be destroyed after matched bits are generated. The finally produced bits approach yields the bit 1 if the SOP is “|” or “⁄” or “±_|” or “Ӿ_/”;\ and 0 if the sop is “—“ or “\” or “±_—” or “Ӿ_\\”. The qubit measurement and bitrate production functions are specified as:

**public int** bitCreate (String sop){     **if** (sop.equals(“|”))        **return** 1;     **else if** (sop.equals(“/”))       **return** 1;     **else if** (sop.equals(“**±_**|”))     **return** 1;     **else if** (sop.equals(“Ӿ_/”))     **return** 1;     **else if** (sop.equals(“—”))       **return** 0;     **else if** (sop.equals(“\\”))      **return** 0;     **else if** (sop.equals(“**±_**—”))   **return** 0;     **else if** (sop.equals(“Ӿ_\\”)) **return** 0;     **else return** null;}**public int qubit**Measure (Qubit Singqubit, String base_){     **int** bitset;     **if** (singqubit.base.equals(base_))     {     bitset = bitCreate(myqubit.sop); singqubit = **null**;     }     **else**     {     singqubit.base= base_;     singqubit.sop = singqubit.createSop(base_);     bitset = bitCreate(singqubit.sop);     singqubit = **null**;     }     **return** bitset;}

To provide more security to encrypt the sensitive information of users, we need to produce a secret quantum key with a reasonable count of qubits. Here, we show the list of methods used to generate a good number of qubits; from these, we extract the final secret key for the encipherment and decipherment process. The first step of the algorithm is that Singh needs to pass a random integer, RI. Here, RI is the count of qubits he plans to produce for the transmission. Recall that we should have a base and SOP for the construction of qubits. Singh initially makes a list of commands by invoking the random base method, and as per that list, he produces his list of SOPs mapped with the list of qubits. Afterward, Singh utilizes both lists to make the final list of mapped qubits with their respective bases, which he will send to King. Later, King will obtain the qubit list of Singh; he utilizes the RI supplied by the count to produce his base list as Singh already has done. King also creates his bit set list with the help of using the qubit measurement process. Now that both Singh and King have their base and bit set lists, both shall interchange their base lists to construe their lists of bit sets. If their occurred bases are matched, then the resultant bit list is used to generate a secret key. Otherwise, it is discarded. The result is a shared secret key, whose size is approximately half of RI. To evaluate this, we maintain the approximate size of the secret key related to RI. For every running instance, the proportion varies and progressively touches just about half of the actual length. In existing models, due to the production of shorter key length and after filtration, the final key is much shorter and is vulnerable to attacks specifically in Social Internet of Things (SIoT) and related applications, blockchain based mobile applications, wireless body area network based applications, computer vision based applications [50], fuzzy optimized techniques, SAE2 based heterogeneous sensitive data [36,50,51,52,53,54,55].

**Figure 3:**
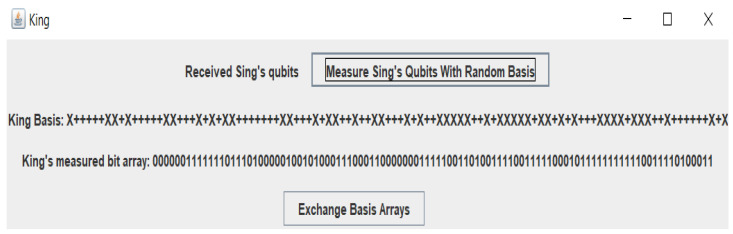
Singh collecting RI, producing a base, SOP, and sharing multi-qubit list to King.

**Figure 4:**
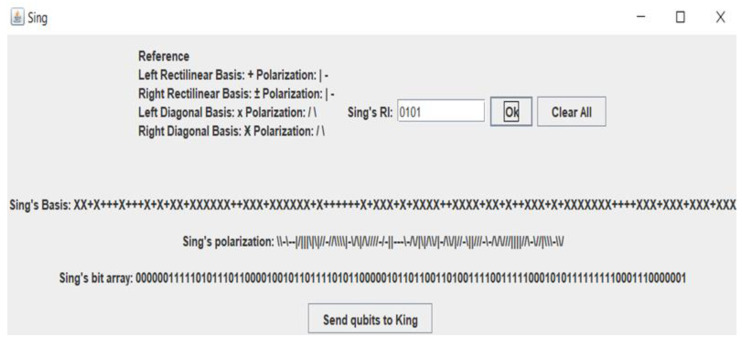
King calculated the multi-qubit list of Singh with his chaotic base list.

**Figure 5:**
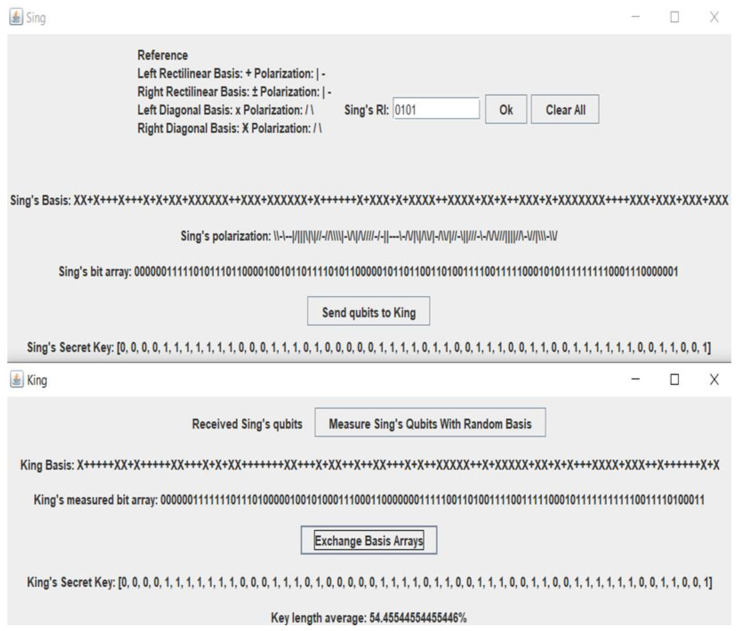
King and Singh interchange base lists and produce a random matched secret key.

Figure 3, Figure 4 and Figure 5 below demonstrate the end-to-end process of the base list and the exchange of SOP among Singh and King with attacks and without attacks.

**Figure 6:**
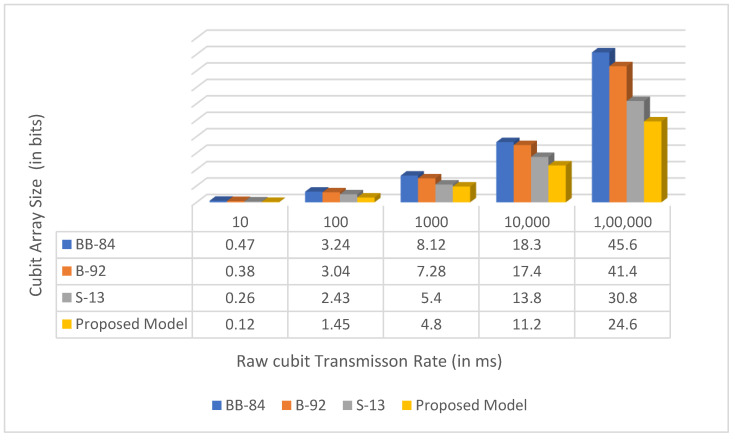
Comparison of the proposed model to the existing models in terms of raw qubit transmission time.

**Figure 7:**
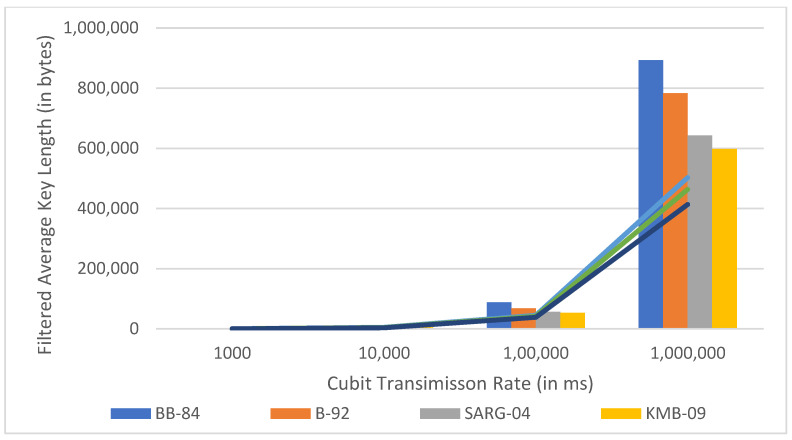
Comparison of the proposed model to the existing models in terms of average key length.

**Figure 8:**
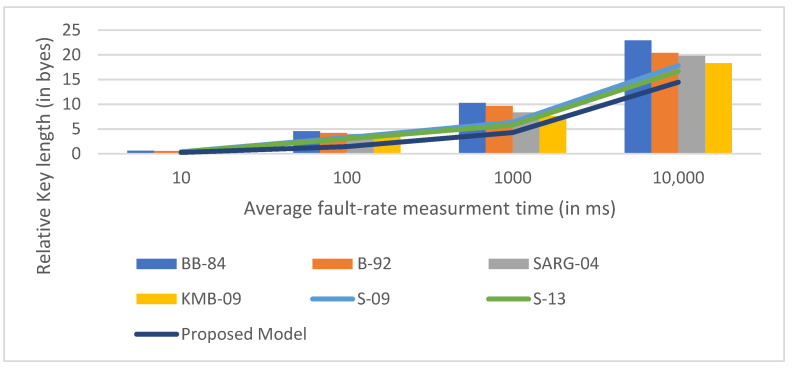
Comparison of the proposed model to the existing models in terms of average fault rate measurement time.

Figure 6, Figure 7 and Figure 8 are the comparative analysis and results of traditional models with our proposed model concerning various parameters such as qubit transmission, measurement rate, average key length, and key exchange time.

## 6. Conclusions and Future Scope

The proposed model is highly chaotic and dynamic. Despite this, it works fine to exhibit the notion and how it performs compared to previous QKD models. In this paper, we only demonstrated how keys are exchanged between IIoT devices in the absence of attacks and theoretically proved that this model provides more security from all types of quantum attacks compared to traditional models. In addition, this model uses less qubit transmission rate and key exchange rate along with minimal computational overhead and shows more efficacy with regards to error-rate measurement to detect whether snooping has happened or not. In future work, we can add an unauthorized person or intruder and demonstrate how to address different advanced quantum attacks at one time while sensitive data are transmitted among multiple IIoT devices. It could be possible to examine various privacy and security parameters of the proposed standard, such as the minimum bit size needed to detect intruders. At the same time, with the communication of qubits among IoT and IIoT devices, we can enhance their security by strengthening the projected model with a multi-photon multi-state entanglement approach using Qiskit for working with quantum computers at the level of pulses, circuits, and IoT and IIoT application modules.

## Figures and Tables

**Table 1:** Comparative analysis of existing QKD approaches.

Parameter	BB84 [33,37]	BB92 [17,38]	SARG04 [27,37]	KMB09 [37,38]	S09 [32,38]	S13 [37,38]	Proposed Model
Number of states	4	2	4	2	Arbitrary states	4	8
Principals	Heisenberg	Heisenberg	Heisenberg	Heisenberg	Public/ private key	Heisenberg	Heisenberg
Polarization	Orthogonal	Non- Orthogonal	Orthogonal	Arbitrary	Bit/phase flip	2 orthogonal	Arbitrary
DoS attack	Vulnerable	Vulnerable	Vulnerable	Vulnerable	N/A (not available)	N/A	N/A
Middle-man attack	Vulnerable	Robust	Robust	Robust	Robust	N/A	N/A
PNS attack	Vulnerable	Vulnerable	Itis better than BB84	Robust	N/A	N/A	N/A
Beam-splitter attack	Vulnerable	Vulnerable	Robust	Robust	N/A	N/A	N/A
Security	Good for long distance	Average	Average	Average	Best for a short distance	Average	Good for both short and long distance
Efficiency	Low	Best	Average	Low	Good	Average	Best

**Table 2:** SOPs of QKD-related qubits and associated properties.

Property (P)	Explanation
P1	Nobody can measure/gain results without disquieting the structure.
P2	Nobody can instantaneously predict the state and impetus of a qubit while transmitting.
P3	Nobody can instantaneously predict the SOP of a qubit in the horizontal-vertical streams.
P4	Nobody can instantaneously predict the SOP of a qubit in the left and right diagonal streams.
P5	Nobody can create a replica, in any instance, of the randomized qubit state.
P6	Nobody can entice the images of qubit transmission practice.

**Table 3:** BB84 qubit SOP-based encrypting design.

Polarizer Angle Base	Polarity Angle	Qubit State Value
Rectilinear (┼)	0°	Zero
	Π/2°	One
Orthogonal (X)	Π/4°	Zero
	3Π/4°	One

**Table 4:** The BB84 QKD design standard.

Stage #	Explanation
Move 1	Singh selects a randomized stream of bits based on the base of a randomized qubit SOP.
Move 2	Singh transmits a randomized stream of bits to King based on a rectilinear-based polarizer and the diagonal-directed polarizer.
Move 3	King obtains the qubits of Singh based on the rectilinear-based polarizer and the diagonal-directed polarizer qubit directions; they measure all the matched patterns using the same basis or a randomized design if King has a different basis than Singh, as shown in Table 2.
Move 4	King declares his measured threshold value of received randomized qubits to Singh. Afterward, King and Singh interchange their estimated randomized bit stream.
Move 5	King and Singh compare the match percentage of their individually measured randomized bit stream. Afterward, they consider the similar qubit patterns they measured on the same basis and discard the randomized bit stream, which they measured with a different basis eliminating respective bits in their bit strings if they measured the photon with another basis.
Move 6	The qubits are equivalent according to their matched measured basis of qubits, and those collections of bits are treated as the secret key for encryption and decryption.

**Table 5:** Photon polarization encoding scheme for BB92.

Polarizer Angle Base	Polarity Angle	Qubit State Value
Rectilinear (“┼”)	0°	Zero
Diagonal (“X”)	Π/4°	One

## Data Availability

Not applicable.
